# Knocking Out *OsRLK7-1* Impairs Rice Growth and Development but Enhances Its Resistance to Planthoppers

**DOI:** 10.3390/ijms241914569

**Published:** 2023-09-26

**Authors:** Shanjie Han, Zhifan Shen, Qing Gao, Nuo Jin, Yonggen Lou

**Affiliations:** 1 State Key Laboratory of Rice Biology and Breeding & Ministry of Agriculture Key Lab of Agricultural Entomology, Key Laboratory of Biology of Crop Pathogens and Insects of Zhejiang Province, Institute of Insect Sciences, Zhejiang University, Hangzhou 310058, China; hanshanjie@zju.edu.cn (S.H.); 3180100231@zju.edu.cn (Z.S.); qinggao@zju.edu.cn (Q.G.); njin@zju.edu.cn (N.J.); 2 Hainan Institute, Zhejiang University, Sanya 572025, China

**Keywords:** receptor-like kinase, rice, jasmonic acid, abscisic acid, herbivore-induced plant defense

## Abstract

Leucine-rich repeat receptor-like kinases (LRR-RLKs) are an important subfamily of receptor-like kinases (RLKs) in plants that play key roles in sensing different biotic and abiotic stress. However, the role of LRR-RLKs in herbivore-induced plant defense remains largely elusive. Here, we found that the expression of a rice gene, *OsRLK7-1*, was induced by mechanical wounding, but was slightly suppressed by the infestation of gravid females of brown planthopper (BPH, *Nilaparvata lugens*) or white-backed planthopper (WBPH, *Sogatella furcifera*). Through targeted disruption of *OsRLK7-1* (resulting in the *ko-rlk* lines), we observed an augmentation in transcript levels of BPH-induced *OsMPK3*, *OsWRKY30*, *OsWRKY33*, and *OsWRKY45*, alongside heightened levels of planthopper-induced jasmonic acid, JA-isoleucine, and abscisic acid in plant tissues. These dynamic changes further facilitated the biosynthesis of multiple phenolamides within the rice plants, culminating in an enhanced resistance to planthopper infestations under both lab and field conditions. In addition, knocking out *OsRLK7-1* impaired plant growth and reproduction. These results suggest that *OsRLK7-1* plays an important role in regulating rice growth, development, and rice-planthopper interactions.

## 1. Introduction

Plants have evolved several mechanisms to withstand diverse biotic and abiotic stresses. Plant defense responses guided by herbivore-associated molecular patterns (HAMPs) are the main mechanisms of plant resistance to herbivores. Several downstream events occur following the recognition of HAMPs, such as the production of reactive oxygen species (ROS), the influx of calcium (Ca^2+^), and the activation of mitogen-activated protein kinase (MPK) cascades [1,2]. These events activate the signaling pathways mediated by phytohormones, including jasmonic acid (JA), salicylic acid (SA), ethylene, and abscisic acid (ABA). These changes lead to the up-regulation of defense-related genes and the biosynthesis of defensive compounds, thereby enhancing the resistance of plants to herbivores [3,4].

Leucine-rich repeat receptor-like kinases (LRR-RLKs) are the largest subfamily of receptor-like kinases in plants [5]. For example, there are approximately 226 LRR-RLKs in *Arabidopsis thaliana* [6] and 332 LRR-RLKs in rice [7]. The LRR-RLKs have three typical structural domains: an extracellular LRR domain, a transmembrane domain, and an intracellular kinase domain. Intracellular kinase structures are activated when the extracellular LRR domain of LRR-RLKs binds to ligands, which leads to the phosphorylation of downstream proteins and the activation of downstream signaling pathways, then regulates various physiological and biochemical processes in plants [8,9]. Thus far, LRR-RLKs have been reported to play an important role in plant growth and development as well as plant responses to abiotic and biotic stresses, including pathogens [10,11,12,13,14]. The flagellin-derived peptide flg22, for instance, induces the development of a monomeric heterodimer of LRR-RLK BAK1 and FLS2 in Arabidopsis, followed by an immune system response [15,16]. In tomato (*Solanum lycopersicum*), the LRR-RLK MRK1 has been identified as a component of the FLS2 and SERK3A/SERK3B complex; the active kinases SlSERK3A and SlSERK3B might be involved in the defense mechanism of tomato against pathogenic bacteria and root-knot nematodes [17]. In rice, the expression of *OsSERK1* can be induced by defense signaling molecules, including SA, JA, and ABA. The overexpression of *OsSERK1* in rice promotes resistance to the rice blast fungus [18]. LRR-RLKs have also been reported to regulate herbivore-induced plant defenses. In maize (*Zea mays*), for example, the LRR-RLK gene *ZmFAC* encodes a receptor that recognizes HAMPs and activates the following defenses [19]. The *OsLRR-RLK1* positively regulates the expression of defense-related genes in rice, promotes the accumulation of JA and ethylene, and enhances the resistance of rice to the striped stem borer (*Chile suppressalis*).

Rice, one of the most important staple crops in the world, is often subjected to infestation by many insect pests, including rice planthoppers, such as brown planthopper (BPH), *Nilaparvata lugens,* and white-backed planthopper (WBPH), *Sogatella furcifera*. Rice planthoppers damage plants directly by sucking phloem sap and laying eggs into plant tissues and indirectly by transmitting viruses; heavy infestation can hinder plant growth and even cause plants’ complete drying and wilting. Previous research has revealed that planthopper infestation alters levels of multiple defense-related phytohormones, including JA, JA-Ile, SA, ethylene, and ABA. Changes in these phytohormones, in turn, enhance the expression of defense-related genes and the production of defensive compounds, such as trypsin protease inhibitors, phenolamides, and volatiles; these responses finally increase the resistance of rice to planthoppers directly and indirectly by attracting the natural enemies of herbivores [20,21]. Recently, it has been reported that BPH infestation induces the expression of *OsLRR-RLK2* and thereby enhances the susceptibility of rice to BPH by decreasing the constitutive phosphorylation level of *OsMPK6* and altering the defense-related phytohormone profile [22]. However, the function of most of rice LRR-RLKs, the largest subfamily of receptor-like proteins in plants, as stated above, in regulating plant resistance to herbivores remains largely unknown.

In this study, we found that a rice LRR-RLK gene, hereafter designated *OsRLK7-1* (*Oryza sativa receptor-like protein kinase 7-1*, *OR472548*), was slightly suppressed by the infestation of gravid BPH females. By combining molecular biology, chemical analysis, reverse genetics, and bioassays, we found that *OsRLK7-1* negatively regulates the biosynthesis of planthopper-induced JA and ABA as well as the resistance of rice to planthoppers.

## 2. Results

### 2.1. The Isolation and Characterization of OsRLK7-1

The full-length cDNA of *OsRLK7-1*, including an open reading frame of 3015 bp, was isolated from a wild-type (WT) variety of rice, Xiushui 11 (XS11). *OsRLK7-1* encodes a protein containing 1005 amino acids with 3 conserved domains: typical leucine-rich repeats, a transmembrane region, and a protein kinase domain (Figure S1). The molecular weight of this protein was predicted to be 105 kDa. Sequence alignment revealed a high similarity (97.75%) to a previously identified rice LRR-RLK gene, *OsRLK7* (also named as *MEG1*) (*Os12g0632800*) [23]; both genes share significant sequence identity in the putative coding region located on chromosome 12 (NC_029267.1, 27109024-27112554). This suggests that *OsRLK7-1* is allelic to *OsRLK7*; their differences are because they come from different varieties (Figure S2). The phylogenetic tree demonstrated that OsRLK7-1 shares high similarities with OgRLK7 in *Oryza glaberrima* (98.90%), OsRLK7 in *Oryza sativa japonica* Nipponbare (97.12%), and ObRLK7 in *Oryza brachyantha* (87.35%) (see Figure S3).

**Figure 1:**
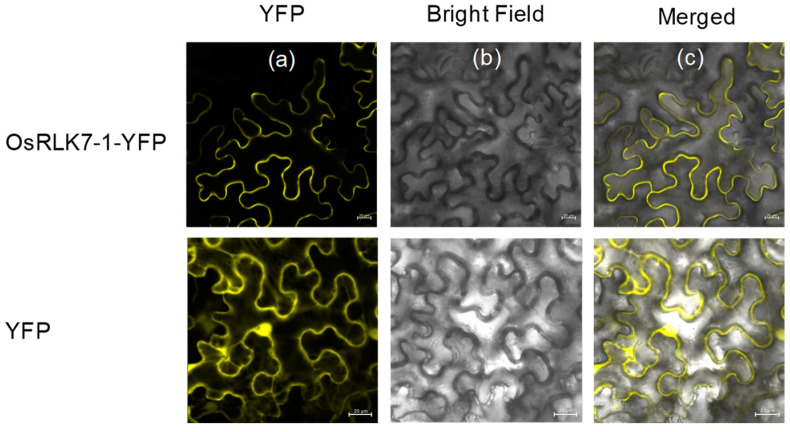
Subcellular localization of OsRLK7-1 protein in tobacco (*Nicotiana benthamiana*) cells. (**a**) Representative confocal microscopy images of tobacco cells expressing *OsRLK7-1*-YFP fusion protein or YFP, with yellow fluorescence indicating the localization of OsRLK7-1 on the plasma membrane. (**b**) Bright field image of the same cells. (**c**) Merged image of (**a**,**b**). Scale bar = 20 μm.

Subcellular localization experiments using a fusion protein (*OsRLK7-1* with yellow fluorescent protein) revealed fluorescent signals emitted from the fusion protein at the plasma membrane (Figure 1). These findings indicated that OsRLK7-1 localizes to the plasma membrane, which might play a role in signal recognition and transduction.

**Figure 2:**
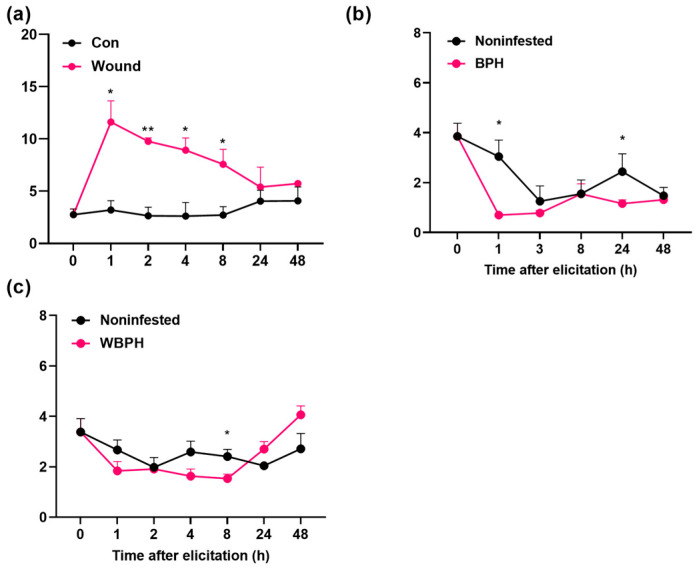
Mean transcript levels (+SE, *n* = 5) of *OsRLK7-1* in leaf sheaths of WT plants that were mechanically wounded (**a**) or infested with gravid BPH females (**b**) or gravid WBPH females (**c**). Con, unmanipulated plants; Noninfested, plants with empty glass cylinders. Asterisks represent significant differences between treatments and controls at each time point (* *p* < 0.05, ** *p* < 0.01, Student’s *t*-tests).

Quantitative real-time PCR (qRT-PCR) revealed that mechanical wounding quickly induced the expression of *OsRLK7-1* in rice (Figure 2a). However, the infestation of gravid females of BPH or WBPH slightly suppressed or did not induce the expression of *OsRLK7-1* (Figure 2b,c).

The qRT-PCR revealed that mechanical wounding quickly induced the expression of *OsRLK7-1* in rice (Figure 2a). However, the infestation of gravid females of BPH or WBPH slightly suppressed or did not induce the expression of *OsRLK7-1* (Figure 2b,c).

### 2.2. Knockout of OsRLK7-1 in Rice

**Figure 3:**
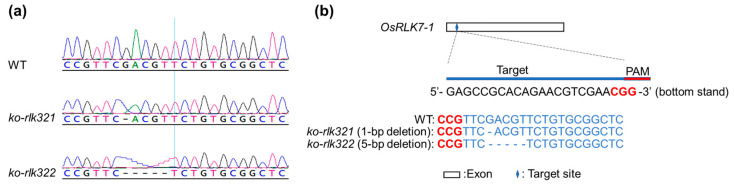
Genotyping of mutations mediated by CRISPR/Cas9 gene editing in the *ko-rlk* lines. (**a**) Sanger sequencing results of WT, *ko-rlk321* and *ko-rlk322*. (**b**) Blue letters, sgRNA targets; blue dashed lines, base deletion; red letters, protospacer-adjacent motif (PAM).

**Figure 4:**
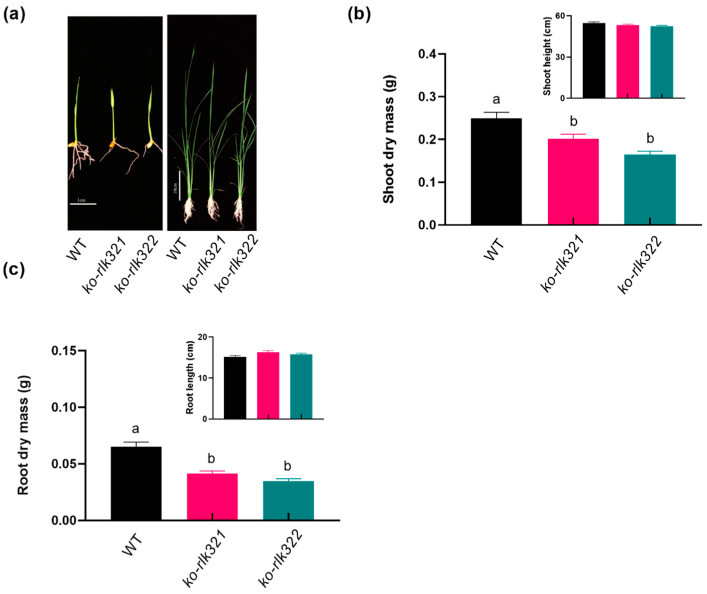
Growth phenotypes of WT and *ko-rlk* plants. (**a**) Photos of 7 d old seedlings (left, bar = 1 cm) and 13 d old seedlings (right, bar = 10 cm) of WT, *ko-rlk321* and *ko-rlk322* lines. (**b**) Mean dry mass (+SE, *n* = 20) of plant shoots. Insert: shoot height. (**c**) Mean dry mass (+SE, *n* = 20) of plant roots. Insert: root length. Different letters indicate significant differences between lines (*p* < 0.05, Tukey’s HSD post hoc test).

Rice lines with the knockout of *OsRLK7-1* (*ko-rlk* lines) were developed using CRISPR/Cas9-mediated gene editing to clarify the function of *OsRLK7-1* in herbivore-induced rice defense responses. We generated two homozygous lines: one with a single G deletion (the 271st nucleotide from the start codon) (*ko-rlk321*) and the other with a GACGT deletion (from the 271st to the 275th nucleotides; *ko-rlk322*, Figure 3a,b). In both lines, the translation of the target sequence terminated prematurely. No mutations of potential off-target sites were found in *ko-rlk* lines (Figure S4). Knocking out *OsRLK7-1* in rice decreased shoot and root dry weight of plants (Figure 4).

### 2.3. OsRLK7-1 Negatively Regulates MPKs and WRKYs

**Figure 5:**
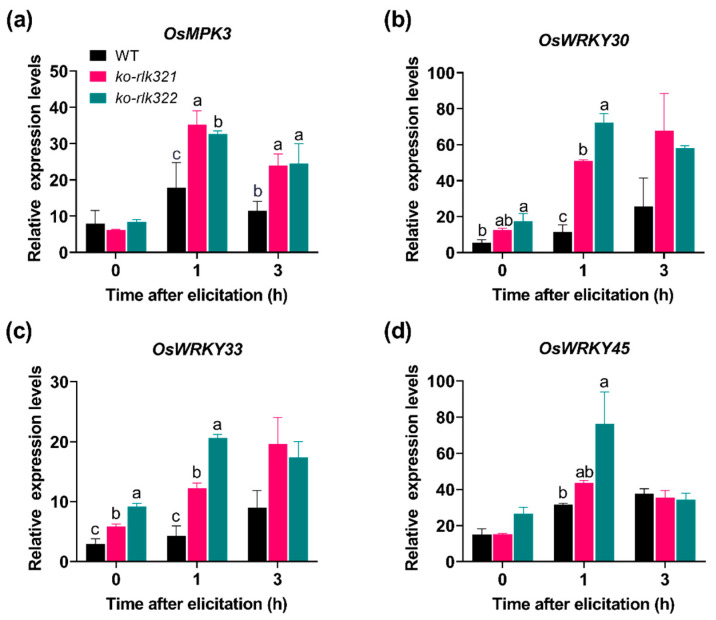
Mean transcript levels (+SE, *n* = 5) of *OsMPK3* (**a**), *OsWRKY30* (**b**), *OsWRKY33* (**c**), and *OsWRKY45* (**d**) in WT and *ko-rlk* plants after they were infested with gravid BPH females. Different letters indicate significant differences among lines at the same time point (*p* < 0.05, Tukey’s HSD post hoc tests).

MPK cascades and WRKY transcriptional factors regulate herbivore-induced defense responses in plants, including rice [24]. In rice, *OsMPK3*, *OsMPK6*, *OsWRKY30*, *OsWRKY33*, and *OsWRKY45* have been reported to be involved in plant defense responses [25,26,27,28,29]. Therefore, we investigated the transcript levels of these MPK and WRKY genes following BPH infestation in both WT plants and the *ko-rlk* lines, *ko-rlk321* and *ko-rlk322*. The results showed that the transcript levels of *OsMPK3*, *OsWRKY30*, *OsWRKY33*, and *OsWRKY45* were significantly higher in *ko-rlk* plants than WT plants (Figure 5). No significant difference in *OsMPK6* transcript levels were observed between *ko-rlk* and WT plants (Figure S5).

### 2.4. OsRLK7-1 Negatively Regulates the Biosynthesis of Induced JA, JA-Ile, and ABA

**Figure 6:**
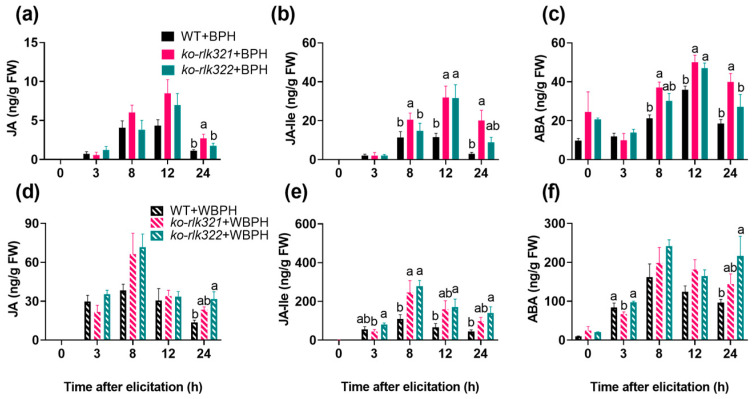
Mean levels (+SE, *n* = 5) of JA, JA-Ile, and ABA in WT and *ko-rlk* plants at different times after they were infested by gravid BPH females (**a**–**c**) or gravid WBPH females (**d**–**f**). Different letters indicate significant differences among lines at the same time point (*p* < 0.05, Tukey’s HSD post hoc tests).

JA-, SA-, and ABA-mediated signaling pathways play an important role in regulating the resistance of rice to herbivores [30,31,32,33]. Thus, we analyzed the JA, JA-Ile, SA, and ABA levels in WT and *ko-rlk* plants before and after BPH and WBPH infestation. No difference was observed in constitutive levels of JA and JA-Ile between WT and *ko-rlk* plants (Figure 6a,b,d,e). However, when plants were infested by gravid BPH or WBPH females, levels of JA (24 h after infestation) and JA-Ile (8–24 h after infestation) were significantly higher in the *ko-rlk* lines, *ko-rlk321* and *ko-rlk322,* than in WT plants (Figure 6a,b,d,e). Knocking out *OsRLK7-1* also did not influence the constitutive level of ABA in rice (Figure 6c,f). However, the planthopper-induced levels of ABA (8–24 h after BPH infestation; 24 h after WBPH infestation) were higher in *ko-rlk* plants than WT plants (Figure 6c,f). Interestingly, regardless of infestation by BPH or WBPH, there was no difference in SA content between WT and *ko-rlk* plants (Figure S6).

### 2.5. Knocking Out OsRLK7-1 Enhances the Phenolamide Synthesis of Planthopper-Induced Rice

**Figure 7:**
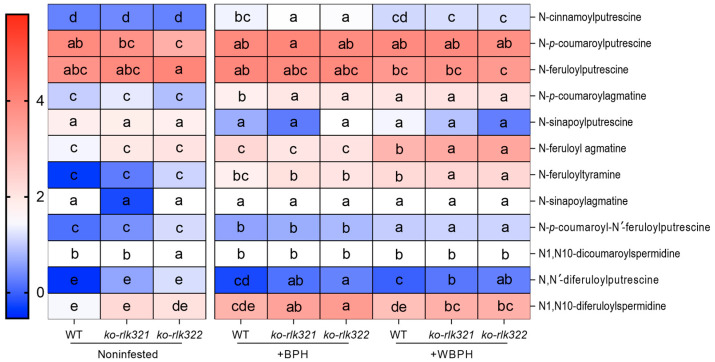
Accumulation of phenolamides in WT and *ko-rlk* plants 0 (Noninfested) and 72 h after they were infested by gravid BPH or WBPH females. Different letters in the same row indicate significant differences among treatments (*p* < 0.05, Tukey’s HSD post hoc tests).

Phenolamides are important defensive compounds against planthoppers in rice [34]. Therefore, we determined the level of 12 different phenolamides in WT and *ko-rlk* plants before and after infestation with BPH and WBPH. In comparison to the wild-type (WT), we observed a significant increase in the accumulation of N-cinnamoylputrescine, N-*p*-coumaroylagmatine, N,N′-diferuloylputrescine, and N1,N10-diferuloylspermidine in the *ko-rlk* plants post injury caused by BPH. Concurrently, there was a noteworthy rise in the levels of N-feruloyl agmatine, N-feruloyltyramine, N, N′-diferuloylputrescine, and N1, N10-diferuloylspermidine when the *ko-rlk* plants were subjected to WBPH infestation (Figure 7).

### 2.6. Knocking Out OsRLK7-1 Enhances the Resistance of Rice to BPH and WBPH

**Figure 8:**
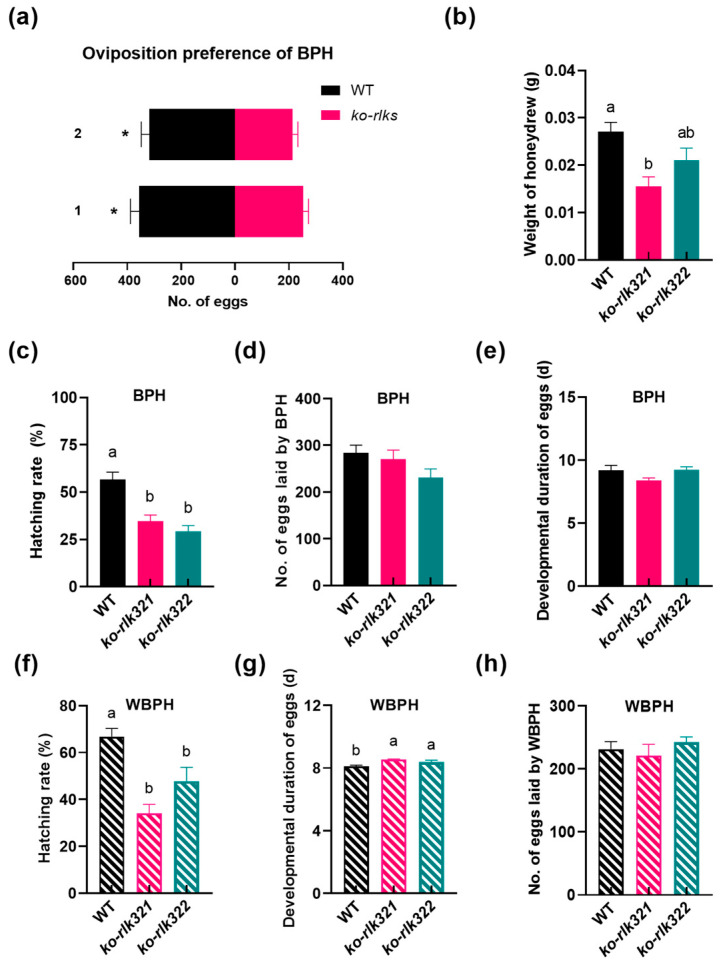
The effect of knocking out *OsRLK7-1* on the performance of BPH and WBPH. (**a**) Mean number (+SE, *n* = 10) of BPH eggs per plant when gravid BPH females were exposed to pairs of plants: 1, WT vs. *ko-rlk321*; 2, WT and *ko-rlk321*. (* *p* < 0.05. Student’s *t*-tests). (**b**) Mean weight (+SE, *n* = 15) of honeydew excreted by a newly emerged BPH adult female for 24 h. (**c**) Mean hatching rate (+SE, *n* = 10) of BPH eggs on WT and *ko-rlk* plants. (**d**) Mean number (+SE, *n* = 10) of BPH eggs on WT and *ko-rlk* plants laid by 15 gravid BPH females for 24 h. (**e**) Mean developmental duration (+SE, *n* = 10) of BPH eggs on WT and *ko-rlk* plants. (**f**) Mean hatching rate (+SE, *n* = 10) of WBPH eggs on WT and *ko-rlk* plants. (**g**) Mean developmental duration (+SE, *n* = 10) of WBPH eggs on WT and *ko-rlk* plants. (**h**) Mean number (+SE, *n* = 10) of WBPH eggs on WT and *ko-rlk* plants laid by 15 gravid WBPH females for 24 h. Different letters illustrate the significant difference between lines (*p* < 0.05, Tukey’s HSD post hoc test).

When BPHs were exposed to pairs of plants (a WT plant vs. a *ko-rlk* plant), BPH preferred to lay eggs on WT plants over *ko-rlk* plants (Figure 8a). Moreover, less honeydew was excreted by BPH when it fed on the two *ko-rlk* plants than when it fed on WT plants (Figure 8b). The hatching rate of BPH eggs was significantly lower on *ko-rlk* plants than on WT plants (Figure 8c). However, no difference was observed in the number of BPH eggs (laid by 15 gravid females for 24 h) and in the developmental duration of BPH eggs between WT and *ko-rlk* plants (Figure 8d,e). In addition, knockout of *OsRLK7-1* also decreased the hatching rate of WBPH eggs and prolonged their developmental duration (Figure 8f,h). There was no difference in the number of eggs laid by 15 gravid WBPH females for 24 h on WT and *ko-rlk* plants (Figure 8g). Knocking out *OsRLK7-1* did not influence the survival rate of BPH and WBPH nymphs, or the developmental duration of BPH and WBPH at the immature stage (Figure S7a–d).

### 2.7. Knocking Out OsRLK7-1 Decreases the Population Density of BPH and WBPH and Rice Yield in the Field

**Figure 9:**
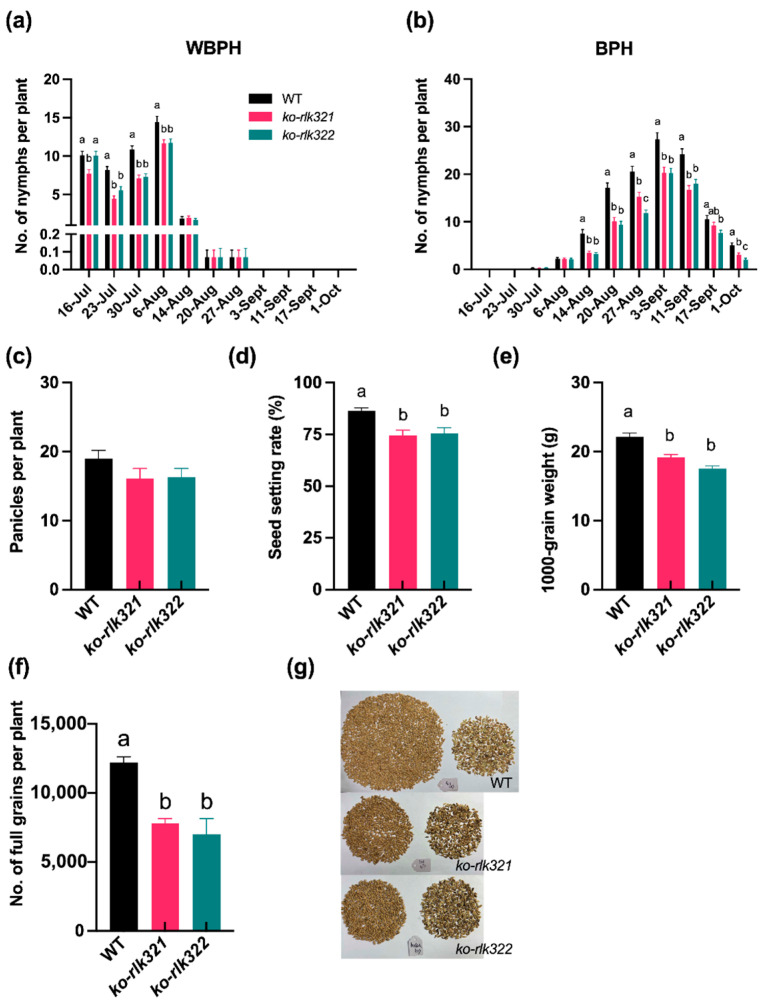
Knocking out *OsRLK7-1* influences rice planthopper resistance and grain yield in the field. (**a**,**b**) Mean number (+SE, *n* = 15) of WBPH nymphs (**a**) and BPH nymphs (**b**) on WT and *ko-rlk* plants at different investigation time points. (**c**) Mean number (+SE, *n* = 10) of panicles per WT or *ko-rlk* plant. (**d**) Mean seed setting rate (+SE, *n* = 10) of WT and *ko-rlk* plants. (**e**) Mean 1000-grain weight (+SE, *n* = 10) of seeds of WT and *ko-rlk* plants. (**f**) Mean number (+SE, *n* = 10) of full grains per WT and *ko-rlk* plants. (**g**) All seeds of WT or *ko-rlk* plant. Left, full seeds; right, shriveled seeds. Different letters illustrate the significant difference between lines (*p* < 0.05, Tukey’s HSD post hoc test).

High population densities of WBPH and BPH were observed on WT compared to *ko-rlk* plants (Figure 9a,b). The population of WBPH peaked on August 8th, with an average of 14.44 nymphs per WT plant and 11.64 and 11.71 nymphs per *ko-rlk* plant, respectively (Figure 9a). BPHs peak population density (September 3rd) was approximately one month later than that of WBPH (Figure 9b). The number of BPH nymphs was 1.35 and 1.36 times higher on WT plants (average of 27.29 nymphs per plant) than on *ko-rlk* plants (20.27 and 20.22).

We also investigated the influence of *OsRLK7-1*-knockout on rice yield. Knocking out *OsRLK7-1* reduced the number of panicles per plant (19.03 on average for WT plants vs. 16.07 and 16.30 on average for *ko-rlk321* and *ko-rlk322* lines) (Figure 9c). There was also a significant reduction in the seed-setting rate in the knockout lines, with an 81.39% rate observed in WT plants, compared to 68.00% and 64.39% in the *ko-rlk321* and *ko-rlk322* lines, respectively (Figure 9d). Furthermore, the 1000-seed weight was found to be lower in the *ko-rlk* lines, with *ko-rlk321* and *ko-rlk322* weighing 19.16 g and 17.54 g, respectively, compared to 22.18 g in the WT plants (Figure 9e). The average yield of the mutant plants was 7784.64 and 7006.33 full grains per plant, yields that were markedly lower than those in the WT plants (12,185.33 in average, Figure 9f,g).

## 3. Discussion

In this study, we found that *OsRLK7-1* encodes a plasma membrane-localized protein (Figure 1). Mechanical wounding strongly and rapidly induced the expression of *OsRLK7-1* (Figure 2a), whereas the infestation of gravid BPH or WBPH females slightly suppressed or did not induce the expression of *OsRLK7-1* (Figure 2b,c). Knocking out of *OsRLK7-1* promoted the expression of *OsMPK3* and several defense-related WRKY genes, and enhanced the accumulation of JA, JA-Ile, and ABA in rice. These changes increased levels of planthopper-induced phenolamides in plants, which in turn improved the resistance of rice to BPH and WBPH. These findings indicated that *OsRLK7-1* plays a key negative regulatory role in defending rice plants against BPH and WBPH.

RLKs have been reported to play an important role in plant defenses, including perceiving herbivore-related signals and influencing the activation of MPK cascades [35,36]. In rice, for instance, *OsLRR-RLK1* responds strongly to herbivory but weakly to mechanical wounding, and it enhances the phosphorylation level and transcript level of herbivore-induced *OsMPK3/OsMPK6* [37]. OsLRR-RLK2, which is induced by the infestation of gravid BPH females, negatively modulates the constitutive activity and transcript level of *OsMPK6* [22]. The homolog of OsRLK7-1 in *Arabidopsis thaliana*, AtRLK7, functions as a receptor of PIP1 (pathogen-associated molecular pattern (PAMP)-induced peptide1, a danger-associated molecular pattern (DAMP)) and plays an important role in amplifying immunity responses triggered by the PAMP flagellin [38]. Hence, the increase in transcript levels of *OsMPK3* in *ko-rlk* lines is probably due to the knockout of *OsRLK7-1* in plants. Future studies should investigate whether *OsRLK7-1* also acts as a receptor of a DAMP like AtRLK7 and whether knocking out *OsRLK7-1* influences the activation of herbivore-induced *OsMPK3*, *OsMPK4*, and *OsMPK6*.

MPK cascades and WRKY transcription factors have been reported to play a central role in plant defense responses via modulating defensive signaling pathways, such as those mediated by JA, SA, ethylene, and ABA [28,29,39]. Moreover, MPKs and WRKYs can regulate each other at the transcriptional and translational levels [25,37,40]. In *Arabidopsis thaliana*, MPK3/MPK6 directly phosphorylate WRKY33, thus regulating SA signaling, ethylene-JA crosstalk, and camalexin biosynthesis [41,42]. In rice, *OsMPK4* positively modulates levels of herbivore-induced SA, JA, and ethylene in plants, which in turn enhance the resistance of rice-to-rice striped stem borer (*Chilo suppressalis* (Walker), SSB) [43]. *OsWRKY30* can be phosphorylated by *OsMPK3/7/14* [44], and then positively regulates rice’s resistance to diseases by affecting JA and SA signaling pathways [28,45]. *OsWRKY53* can physically interact with *OsMPK3/6* and suppress their activity, thereby regulating the herbivore-induced activity of other WRKYs and signaling pathways mediated by SA, JA, and ethylene [46]. In this study, the expression of *OsMPK3*, *OsWRKY30*, *OsWRKY33*, and *OsWRKY45* was significantly higher in *ko-rlk* plants than in WT plants following exposure to BPH (Figure 5). Therefore, the increase in planthopper-induced JA, JA-Ile, and ABA levels in *ko-rlk* plants (Figure 6) might be related to the regulation of *OsRLK7-1* on these MPK cascades and WRKY genes.

The JA- and ABA-mediated signaling pathways play a central role in regulating the resistance of rice to BPH and WBPH [47,48]. The activation of the ABA-mediated pathway in rice, for example, decreases BPH feeding by inducing callose deposition in plant phloem [49,50]. Moreover, the ABA pathway also reduces the hatching rate of BPH eggs [50]. JA signaling pathway plays an important but variable role in mediating the resistance of rice to BPH [51]. However, it seems consistent that the activation of JA pathway reduces the hatching rate of BPH eggs on plants, thereby promoting rice resistance to BPH eggs. For instance, the hatching rate of BPH eggs was significantly higher on rice lines with the knockout of *OsAOC*, *OsOPR7*, or *OsMYC2* (which are critical genes in JA biosynthesis or signaling pathway) than WT plants [30,51]. Hence, the enhanced resistance to both BPH and WBPH observed in *ko-rlk* plants can be primarily attributed to their higher levels of JA, JA-Ile, and ABA compared to WT plants. These findings underscore the interplay between phytohormone signaling and pest resistance in rice and provide valuable insight into the potentially multifaceted mechanisms underlying the observed resistance in *ko-rlk* plants. Future research should delve deeper into the specific components within the JA and ABA pathways that are modulated by *OsRLK7-1*. Moreover, it is also imperative to explore the involvement of additional signaling pathways in the regulation of resistance mechanisms in *ko-rlk* lines, as well as investigate potential crosstalk between these pathways.

In rice, phenolamides are important defensive compounds against herbivores, and their biosynthesis is regulated by the JA signaling pathway [37,52,53]. We found that levels of six phenolamides (N-cinnamoylputrescine, N-*p*-coumaroylagmatine, N,N′-diferuloylputrescine, N1,N10-diferuloylspermidine, N-feruloyl agmatine, and N-feruloyltyramine) were significantly higher in BPH or WBPH-infested *ko-rlk* plants than in similarly treated WT plants (Figure 7). Among these six phenolamides, N-feruloyltyramine and N1, N10-diferuloylspermidine have been reported to reduce the survival rate of WBPH [48]. Hence, higher levels of phenolamides in *ko-rlk* plants compared to WT plants might explain the increased resistance in *ko-rlk* plants to BPH and WBPH. Future research should elucidate the involvement of additional defensive compounds in *OsRLK7*-mediated resistance in rice in order to provide a more comprehensive understanding of the interaction between rice plants and herbivores. Our investigation also revealed notable distinctions in the morphological attributes between *ko-rlk* plants and WT plants. Compared to WT plants, *ko-rlk* plants exhibited lighter roots and shoots. The decrease in plant biomass was, notably, accompanied by a significant reduction in the grain yield of *ko-rlk* plants (Figure 9d–g). These observations are consistent with the results of previous studies suggesting that *OsRLK7* plays a key role in regulating grain filling and thereby influences the 1000-seed weight of plants [23]. The results demonstrate that *OsRLK7-1* is required for rice growth and reproduction. Our ongoing and future research endeavors are poised to delve deeper into the underlying mechanisms governing these specific effects.

In conclusion, our findings demonstrate that rice can recognize the infestation of planthoppers and then enhance plant resistance by suppressing the expression of *OsRLK7-1*. Moreover, like most defense-related pathways or components, such as the JA-mediated signaling pathway and *OsWRKY70*, which positively regulate plant herbivore resistance but negatively mediate plant growth [3,54], the increased herbivore resistance in rice mediated by *OsRLK7-1* also occurs at the expense of plant growth and reproduction. Elucidating the mechanisms underlying *OsRLK7-1* regulation of rice growth and reproduction would provide a basis for breeding new rice varieties that are resistant to herbivores as well as not harmful to plant growth.

## 4. Materials and Methods

### 4.1. Plant Growth and Insects

The WT rice (*Oryza sativa* subsp. *japonica*) variety XS11 and mutants *ko-rlk321* and *ko-rlk322*, which were derived from XS11, were used in experiments. Seven days after germination, seedlings were cultivated in 20 L hydroponic boxes (51 × 35 × 17 cm) with rice nutrient solution as described previously [29] (Figure S8a). At 30 days of age, they were individually transferred to single 250 mL plastic cups (mouth diameter 7 cm, bottom diameter 5 cm, height 10 cm) (Figure S8b) for subsequent experiments. All plants were kept in a growth chamber at 28 ± 2 °C and 80 ± 10% humidity under a 14 h/10 h light/dark photoperiod, light intensity, 5500 ± 200 Lx.

BPH and WBPH were collected from rice fields in Hangzhou, China, and reared on rice seedlings of a susceptible rice variety, Taichong Native 1, in an artificial climate chamber at 26 ± 2 °C and 60 ± 10% humidity under a 14 h/10 h light/dark photoperiod, light intensity, 4500 ± 200 Lx.

### 4.2. Plant Treatment

For mechanical wounding, the lower parts of individual plant shoots were punctured 200 times using a No. 3 insect needle (40 mm long and 0.45 mm in diameter). Unmanipulated plants were used as controls. For BPH or WBPH infestation, individual plant shoots were confined in glass cylinders (diameter 4 cm, height 8 cm, with small holes, Figure S8c) into which 15 gravid females of BPH or WBPH were introduced. Plants with empty glass cylinders were used as controls.

### 4.3. RNA Extraction and Quantitative Real-Time PCR (qRT-PCR)

RNA was isolated using the FastPure^®^ Universal Plant Total RNA Isolation Kit (Vazyme, Nanjing, China). Reverse transcription was conducted using the Hiscript^®^ II Q RT Supermix for qPCR (+GDNA WiPer) (Vazyme). The qRT-PCR was performed on a CFX96™ Real-Time PCR System (Bio-Rad, Hercules, CA, USA) using the Taq Pro Universal SYBR qPCR Master Mix (Vazyme). Refer to the product instruction manual for all operating procedures in this section. The −2^ΔΔCt^ method was employed for comparative gene expression analysis, with *OsACTIN* (TIGR ID: *LOC_Os03g50885*) used as an internal standard. The primer sequences used in qRT-PCR are listed in Table S1.

### 4.4. Cloning of OsRLK7-1 and Sequence Analysis

The full-length cDNA of *OsRLK7-1* was obtained by reverse transcription (RT)-PCR from total RNA isolated from WT plants using a pair of primers (forward primer: 5′-ATGCCACCGCCCTCCCTTCT-3′; reverse primer: 5′-CTAGGGGATCACCTTGACCTTCACCGA-3′) (Table S2). The PCR was performed with KOD One^TM^ PCR Master Mix (TOYOBO, Shanghai, China), and the products were cloned into the pEASY^®^-Blunt Simple Cloning Vector (TransGen Biotech Co., Ltd. Beijing, China) and transformed into component cell DH5α. Selection of transformed cells was facilitated by growth on selective media containing ampicillin. Plasmid DNA was isolated from positive clones, and the inserted cDNA was verified by sequencing. The software program, Molecular Evolutionary Genetics Analysis (MEGA, version 11.0.13), was employed to construct a phylogenetic tree for *OsRLK7-1* and its homologs from other plant species using the neighbor-joining method with 1000 bootstrap replicates. The protein sequences of *OsRLK7-1*, and its homologs from different plant species were downloaded from the National Center for Biotechnology Information (NCBI, http://www.ncbi.nlm.nih.gov, accessed on 6 January 2023).

### 4.5. Subcellular Localization Assay

The full-length coding sequence of *OsRLK7-1* without the stop codon was PCR amplified from total RNA isolated from WT plants using a pair of primers (forward primer: 5′-ACGCGTTTAATTAAGATGCCACCGCCCTCCCTTCT-3′; reverse primer: 5′-TGACGTCCCGGGATCCGGGGATCACCTTGACCTTCACCGA-3′). The PCR product was cloned into the pBA002-YFP vector by 2 × Seamless Cloning Mix (Biomed, Beijing, China), resulting in the fusion of *OsRLK7-1* with the YFP reporter gene. The resulting construct was transformed into DH5α, and positive transformants were selected and verified by sequencing. Subsequently, the constructed pBA002-YFP-*OsRLK7-1* vector (Figure S9a) was introduced into *Agrobacterium tumefaciens* strain GV3101 via electrotransformation. The vector was then transiently expressed in tobacco (*Nicotiana benthamiana*) leaves via *Agrobacterium tumefaciens*-mediated transformation (GV3101, 10 mM MES and MgCl_2_, 150 µM acetosyringone, pH = 5.6) [37]. After 2 d of infiltration, Zeiss LSM 800 Confocal Laser Scanning Microscope (Carl Zeiss Microscopy GmbH, Jena, Germany) was used for fluorescence detection. Primers used in the analyses are listed in Table S2.

### 4.6. Generation of Rice Lines with OsRLK7-1 Knocked Out

Rice lines with knocked-out *OsRLK7-1* were obtained using CRISPR/Cas9-mediated gene editing. The online CRISPR-GE tool (http://skl.scau.edu.cn/home/, accessed on 18 March 2019) [55] was employed to design the target sequence of *OsRLK7-1*. The target sequence (5′-GAGCCGCACAGAACGTCGAA-3′) was cloned into a sgRNA vector to yield OsU6b promotor-deriven single-guide RNA (sgRNA) expression cassette by overlapping PCR [56]. The sgRNA expression cassette was amplified by PCR using site-specific primers (Pps, Pgs), digested by *Bsa I* and then integrated into plant CRISPR-Cas9 binary vector pYLCRISPR/Cas9Pubi-H (Figure S9b). The vector was used for transforming rice variety XS11 using an *A. tumefaciens*-mediated transformation system. Plants with mutations but lacking the T-DNA were screened by target DNA sequencing and hygromycin resistance. Two homozygous T_2_ lines with *OsRLK7-1* knocked out, *ko-rlk321* and *ko-rlk322*, were used for all experiments. Off-target site prediction was conducted using CRISPR-GE; since all off-target scores were exceptionally low (<0.05), we selected the three top-scoring sites (intronic and intergenic regions were excluded) for primer design and Sanger sequencing for *ko-rlk* lines and WT plants. Primer sequences used for vector construction and off-target testing are provided in Table S2.

### 4.7. BPH and WBPH Bioassays

To investigate the effect of knocking out *OsRLK7-1* on the oviposition preference of BPH, pots with two plants (one WT, one *ko-rlk* plant) were individually confined in the glass cylinders. Fifteen BPH gravid females were introduced into each pot (Figure S8c). After 24 h, the number of eggs on each plant was counted under a stereoscope [29]. Ten replicates were used in this experiment.

To determine the effect of *OsRLK7-1* on BPH feeding, we measured the amount of honeydew excreted individually by newly emerged BPH female adults for 24 h on WT or *ko-rlk* plants. One newly emerged female adult was carefully introduced into a triangular parafilm bag (5.7 × 4 × 4 cm) and then the bag was securely attached to a rice shoot (BPH could reach the plant and feed on it) (Figure S8d). The mass of honeydew excreted was measured after 24 h [29]. Fifteen replicates were used in this experiment.

To measure the effect of *OsRLK7-1* on the hatching rate of BPH and WBPH eggs, 10 gravid BPH females were allowed to lay eggs on individual WT and *ko-rlk* plants for 24 h. After removing all of the females, the newly hatched nymphs were counted and removed daily until no new nymphs were found. The unhatched eggs on each plant were counted under a stereoscope [29]. Ten replicates were performed in this experiment.

To explore the effect of knocking out *OsRLK7-1* on the survival rate and developmental duration of BPH or WBPH nymphs, WT and *ko-rlk* plants were individually covered with the glass cylinders into which 15 newly hatched nymphs were released. The number of surviving nymphs on each plant was recorded daily until 17 d after the release of nymphs. When all nymphs became adults, the developmental duration of the immature stage of BPH and WBPH was also calculated [29]. Fifteen replicates were performed in this experiment.

### 4.8. Phytohormone Analysis

Plants of WT and *ko-rlk* lines were randomly assigned to BPH, WBPH, and non-infested treatments. The outermost two leaf sheaths of individual plants were harvested 0, 3, 8, 12, and 24 h after BPH or WBPH infestation. Samples (each about 0.1 g) were ground in liquid nitrogen, and SA, JA, JA-Ile, and ABA in samples were extracted with ethyl acetate containing internal standards (^2^D6-JA, ^2^D6-JA-Ile, ^2^D4-SA, and ^2^D6-ABA). The concentrations of these hormones were analyzed via high-performance liquid chromatography/mass spectrometry/mass spectrometry (HPLC/MS/MS) using the same method as described in [37]. Five replicates at each time point for each treatment were performed.

### 4.9. Phenolamides Measurements

Plants of WT and two transgenic lines (*ko-rlk321*, *ko-rlk322*) were randomly assigned to BPH, WBPH, and non-infested treatments. The outermost two-leaf sheaths of individual plants were harvested 72 h after BPH infestation. Samples (each about 0.1 g) were ground in liquid nitrogen and the phenolamides in samples were extracted with 70% methanol. The phenolamides were analyzed and quantified using HPLC/MS/MS using the same method as described previously [37]. Five replicates were performed for each treatment.

### 4.10. Field Experiments

A field experiment was conducted in Changxing, Zhejiang, China, from the summer to autumn of 2022. The experimental plot was divided into nine blocks (6 × 5 m), and each block was encompassed by a 1 m rice buffer zone. These blocks were randomly assigned to three lines (two *ko-rlk* lines and one WT line), each line with three replications. Rice seeds were sowed in May 2022, and seedlings were transplanted to the blocks one month later. The number of nymphs and adults of BPH and WBPH were recorded weekly from 16 July 2022 to 1 October 2022, by randomly sampling 15 hills of plants in each block using the following method: WBPHs and BPHs on above-ground parts of each hill of plants were collected into a plastic tray (length 45 cm × width 33 cm × depth 0.8 cm) by softly tapping plants and then they were counted. To measure rice yield, 10 hills of completely mature plants in each block were harvested, and the number of panicles per hill, number of full grains per plant, seed setting rate, and 1000 seed weight were recorded.

### 4.11. Data Analysis

The data were statistically analyzed using Student’s *t*-tests and one-way ANOVA followed by Tukey’s HSD post hoc tests (SPSS software, version 22.0; IBM Corp., Armonk, NY, USA). All figures and graphical representations were constructed using GraphPad Prism version 9 (GraphPad Software, San Diego, CA, USA).

## Data Availability

The data presented in this study are available on request from the corresponding author.

## Supplementary Materials

The following supporting information can be downloaded at: https://www.mdpi.com/article/10.3390/ijms241914569/s1.
