# Supplementary material for: Knocking Out OsRLK7-1 Impairs Rice Growth and Development but Enhances Its Resistance to Planthoppers
Source: Int J Mol Sci. 2023 Sep 26;24(19):14569. doi: 10.3390/ijms241914569 (PMC10572756; doi:10.3390/ijms241914569)
Supplement: Supplementary file 1 [file ijms-24-14569-s001.zip › ijms-2612904-supplementary.pdf]

## Supplementary data

# Knocking Out *OsRLK7-1* Impairs Rice Growth and Development but Enhances Its Resistance to Planthoppers

**Shanjie Han[1], Zhifan Shen[1], Qing Gao[1], Nuo Jin and Yonggen Lou[1,2,*]**

[1] State Key Laboratory of Rice Biology and Breeding & Ministry of Agriculture Key Lab of Agricultural Entomology, Key Laboratory of Biology of Crop Pathogens and Insects of Zhejiang Province, Institute of Insect Sciences, Zhejiang University, Hangzhou 310058, China; hanshanjie@zju.edu.cn(S.H.); 3180100231@zju.edu.cn(Z.S.); qinggao@zju.edu.cn(Q.G.); njin@zju.edu.cn (N. J.)

[2] Hainan Institute, Zhejiang University, Sanya 572025, China

* Correspondence: yglou@zju.edu.cn; Tel.: 0086-571-88982622

**Figure S1.** Sequences of nucleotides and deduced amino acids of *OsRLK7-1.*

**Figure S2.** Alignment of nucleotide sequences of *OsRLK7* and *OsRLK7-1*.

**Figure S3.** Phylogenetic analysis of OsRLK7-1 and its orthologs from different plant species.

**Figure S4.** Sanger sequencing results of potential off-target sites.

**Figure S5.** Mean transcript levels (+SE, n = 5) of *OsMPK6* in WT and *ko-rlk* plants 0, 1 and 3 h after they were infested with gravid BPH females.

**Figure S6.** Mean content (+SE, n = 5) of SA in WT and *ko-rlk* plants at 0, 3, 8, 12 and 24 h after infestation of gravid BPH females (a) or gravid WBPH females (b).

**Figure S7.** Knocking out *OsRLK7-1* does not influence the survival and development of BPH and WBPH nymphs.

**Figure S8.** Experimental setups for plant growth and herbivore bioassays.

**Figure S9.** Vectors used in this study.

**Table S1.** Primers used for qRT-PCR.

**Table S2.** Primers used for PCR.

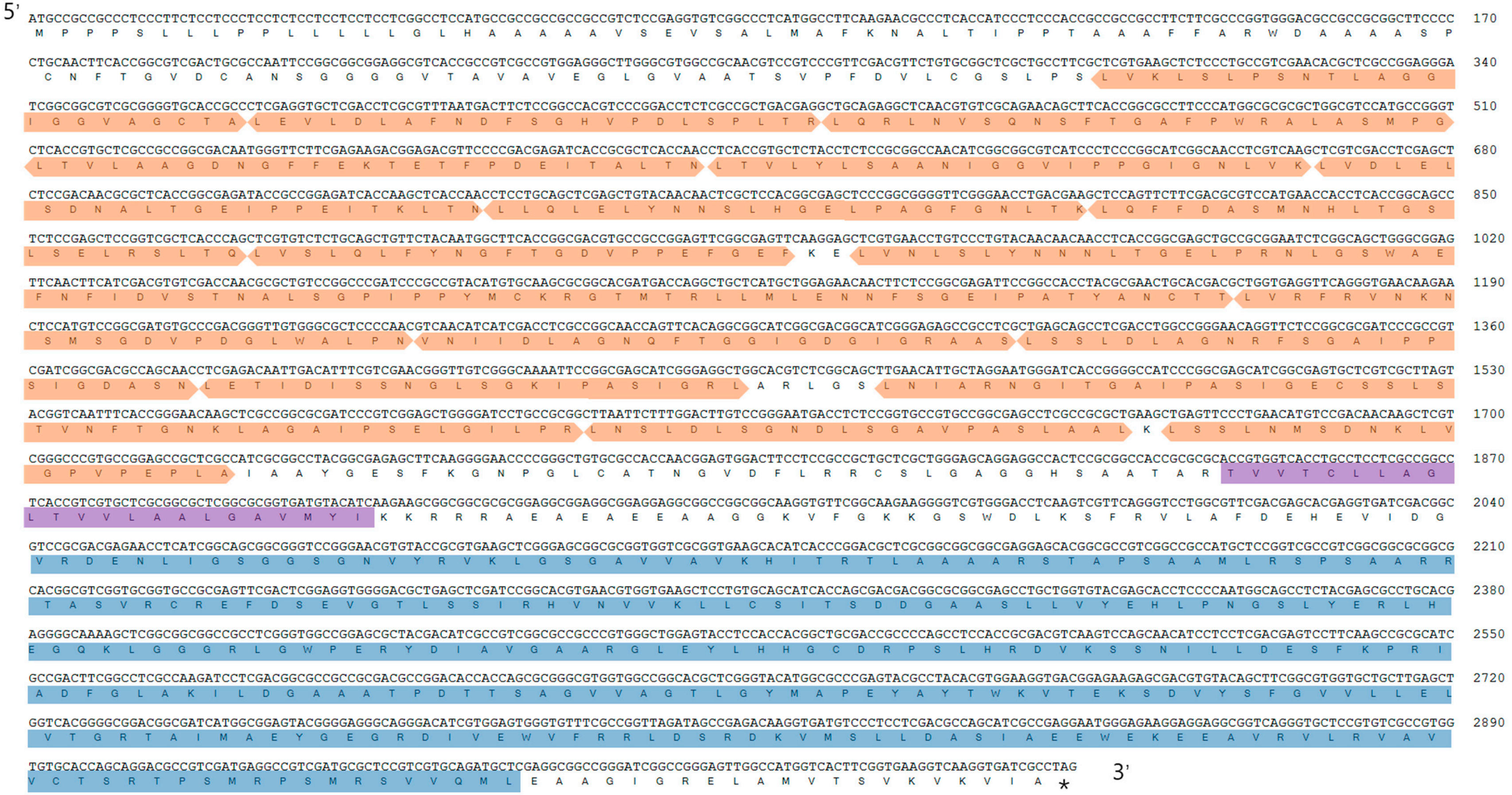


**Figure S1.** Sequences of nucleotides and deduced amino acids of *OsRLK7-1*.

The predicted leucine-rich repeats motifs are in orange; the transmembrane region is in purple; domain of kinase is in blue; and terminator is marked with an

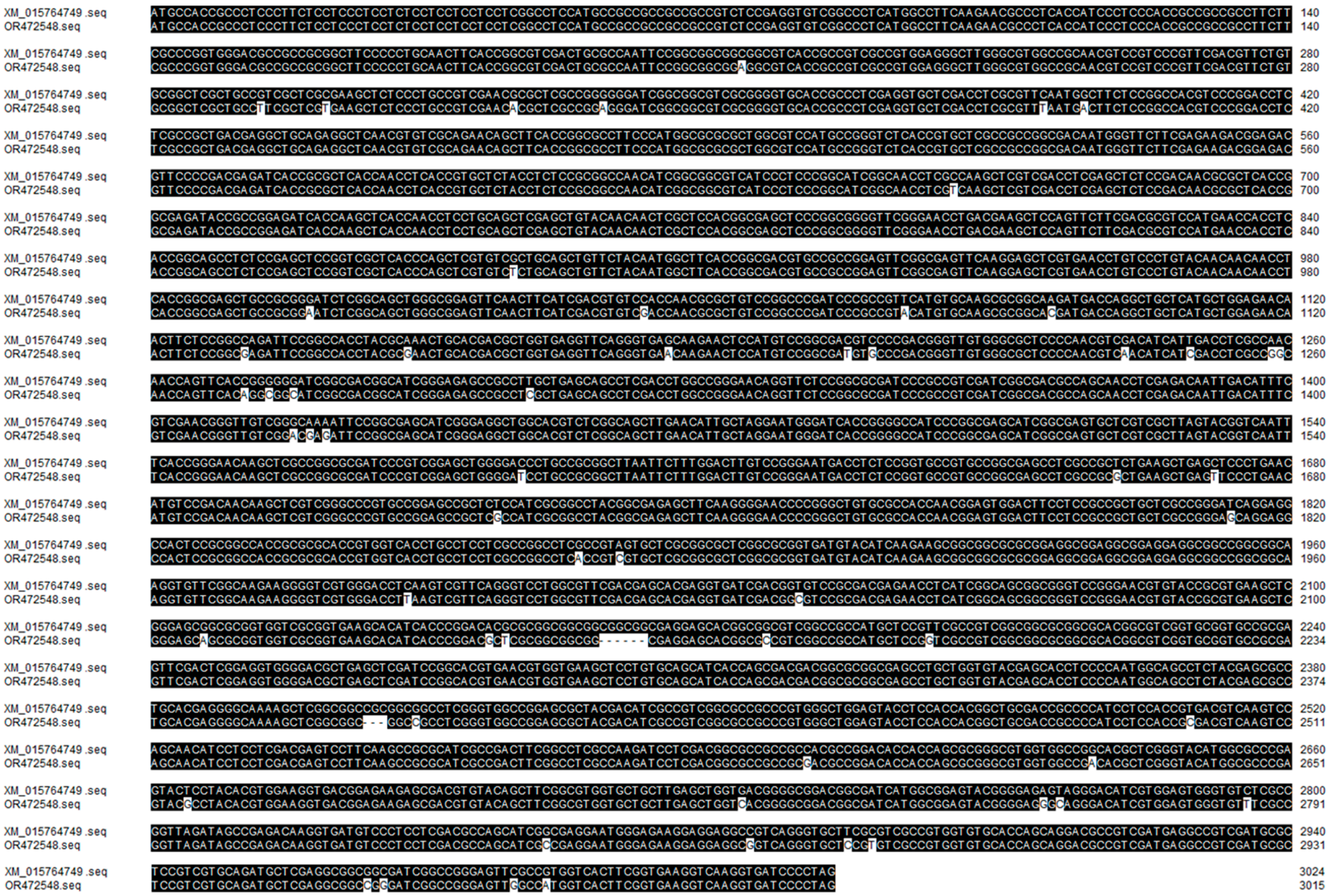


asterisk.

**Figure S2.** Alignment of nucleotide sequences of *OsRLK7* (XM_015764749) and *OsRLK7-1* (OR472548).

Shade (with solid black) indicates identical bases; dashed line, base deletion. The numbers to the right of each line indicate the position of the rightmost base in each line.

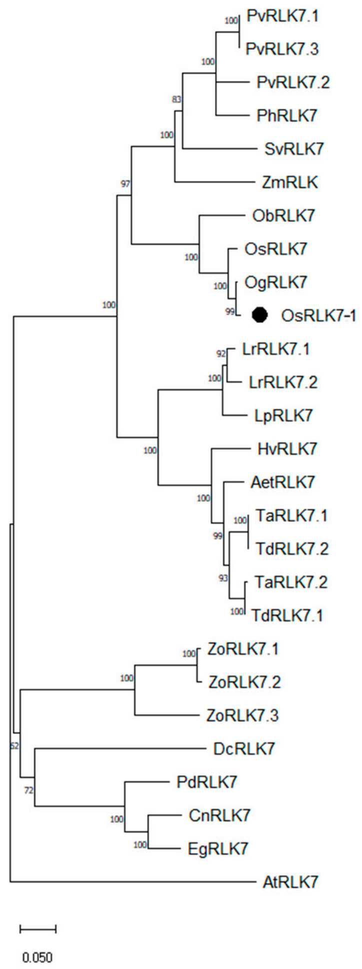


**Figure S3.** Phylogenetic analysis of OsRLK7-1 and its orthologs from different plant species.

The unrooted tree was constructed with a neighbor-joining method based on the alignment of protein sequences using MEGA11 software (default settings except the replicates of the bootstrap value, 1,000 replicates). The bootstrap values for the branches are shown. Plant species acronyms are included before its protein name: Os, *Oryza sativa*; Aet, *Aegilops tauschii* subsp. *strangulate*; At, *Arabidopsis thaliana*; Cn, *Cocos nucifera*; Dc, *Dioscorea cayenensis* subsp. *rotundata*; Eg, *Elaeis guineensis*; Hv, *Hordeum vulgare* subsp. *vulgare*; Lp, *Lolium perenne*; Lr, *Lolium rigidum* ; Ob, *Oryza brachyantha*; Og, *Oryza glaberrima*; Pd, *Phoenix dactylifera*; Ph, *Panicum hallii*; Pv, *Panicum virgatum*; Sv, *Setaria viridis*; Ta, *Triticum aestivum*; Td, *Triticum dicoccoides*; Zm, *Zea mays*; Zo, *Zingiber officinale*. Sequence data in the phylogenic tree can be found in the GenBank with accession numbers as below: OsRLK7-1(OR472548), OsRLK7(XP_015620235.1), AetRLK7(XP_020194737.1), AtRLK7(NP_850942.1), CnRLK7(KAG1348101.1), DcRLK7(XP_039139905.1), EgRLK7(XP_010943231.1), HvRLK7(XP_044948214.1), LpRLK7(XP_051209176.1), LrRLK7.1(XP_047050302.1), LrRLK7.2(XP_047050623.1), ObRLK7(XP_040385827.1), OgRLK7(XP_052139509.1), PdRLK7(XP_038970452.1), PhRLK7(XP_025808423.1), PvRLK7(XP_039840382.1), PvRLK7.1(XP_039803010.1), PvRLK7.2(XP_039803012.1), SvRLK7(XP_034586844.1), TaRLK7.1(XP_044386491.1), TaRLK7.2(XP_044379179.1), TdRLK7.1(XP_037439029.1), TdRLK7.2(XP_037431991.1), ZoRLK7.1(XP_042463340.1), ZoRLK7.2(XP_042427680.1), ZoRLK7.3(XP_042416481.1), ZmRLK(ONM32713.1).

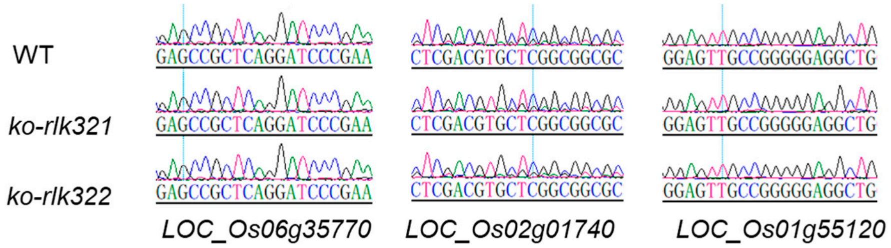


**Figure S4.** Sanger sequencing results of potential off-target sites.

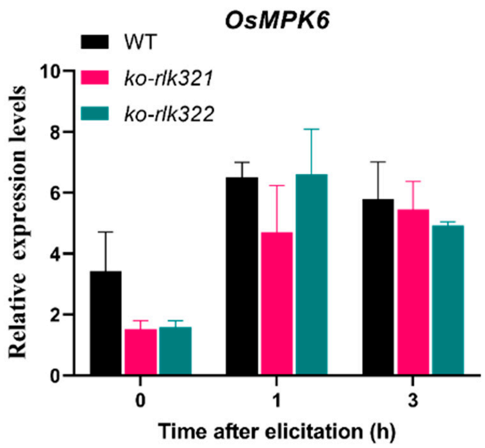


**Figure S5.** Mean transcript levels (+SE, n = 5) of *OsMPK6* in WT and *ko-rlk* plants 0, 1, and 3 h after they were infested with gravid BPH females.

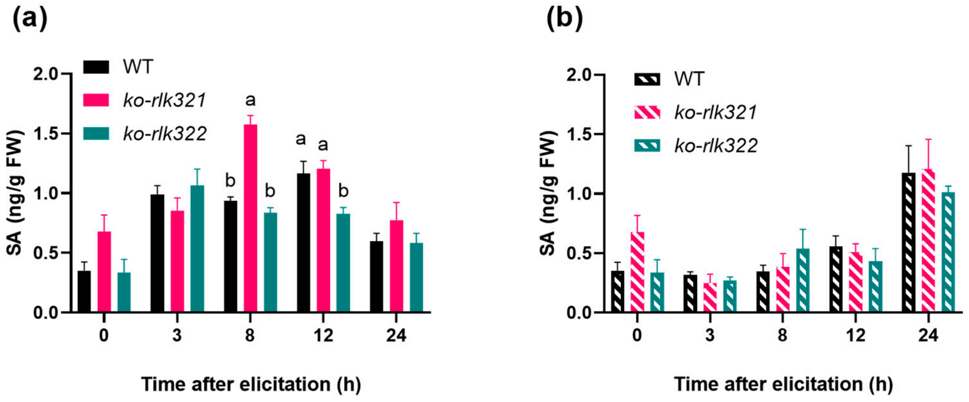


**Figure S6.** Mean content (+SE, n = 5) of SA in WT and *ko-rlk* plants at 0, 3, 8, 12, and 24 h after infestation of gravid BPH females (a) or gravid WBPH females (b). Different letters indicate significant differences among lines at the same time point ($p < 0.05$, Tukey's post-hoc test)..

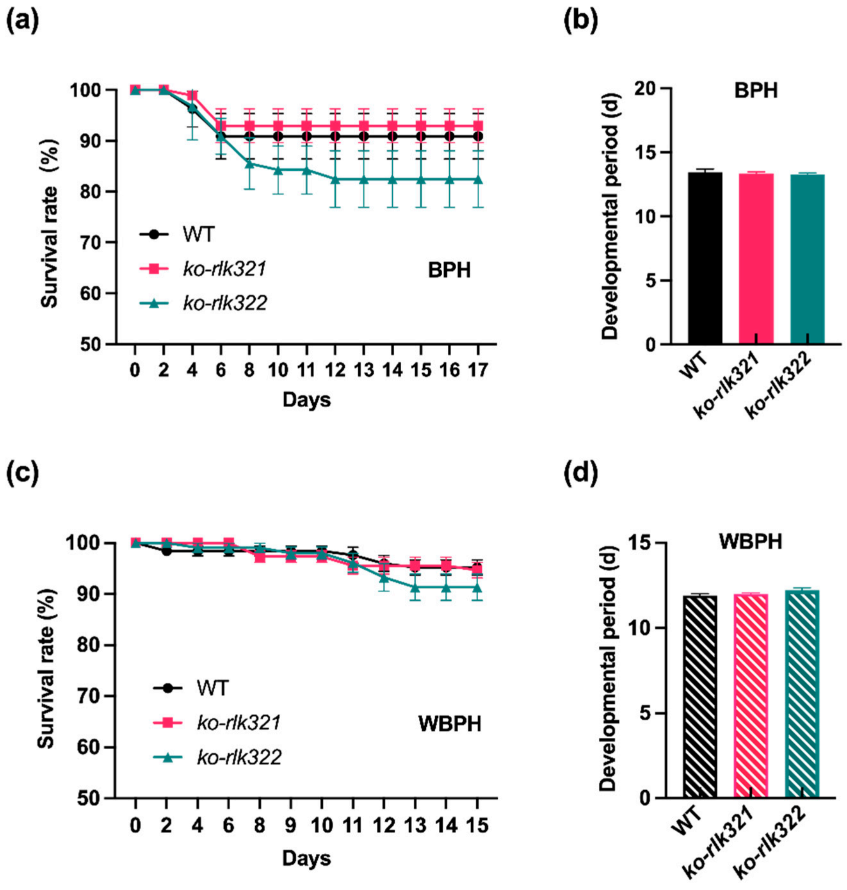


**Figure S7.** Knocking out *OsRLK7-1* does not influence the survival and development of BPH and WBPH nymphs.

(a) Mean survival rate (±SE, n = 15) of newly hatched BPH nymphs on WT and *ko-rlk* (*ko-rlk321* and *ko-rlk322*) plants 0-17 d after they were released. (b) Mean developmental duration of (+SE, n = 15) immature stage of BPH on WT and *ko-rlk* plants. (c) Mean survival rate (±SE, n = 15) of newly hatched WBPH nymphs on WT and *ko-rlk* (*ko-rlk321* and *ko-rlk322*) plants 0-15 d after they were released. (d) Mean developmental duration (+SE, n = 15) of immature stage of WBPH on WT and *ko-rlk* plants.

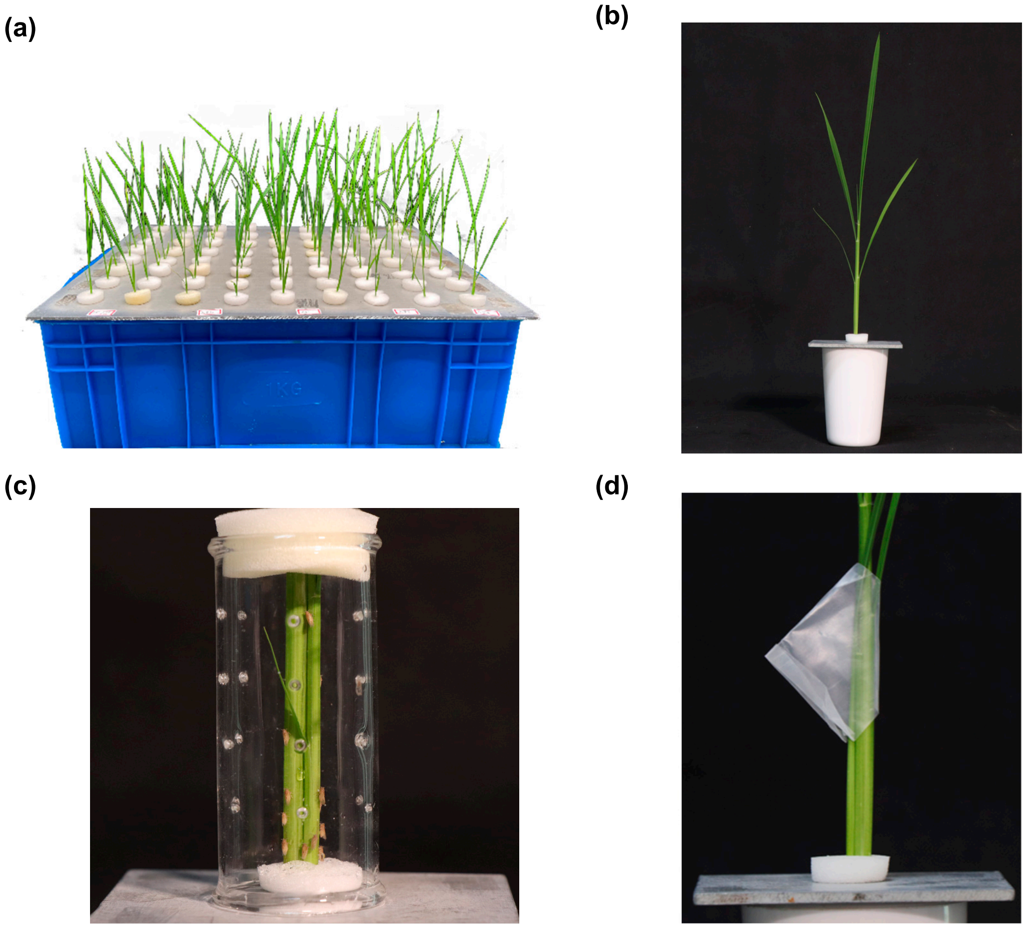


**Figure S8.** Experimental setups for plant growth and herbivore bioassays.

(a) Rice seedlings were planted in a hydroponic box with hydroponic solution. (b) Rice seedlings were individually transferred into single plastic cups. (c) The setup for planthopper bioassays. (d) The setup for planthopper feeding bioassays.

(a)

(b)

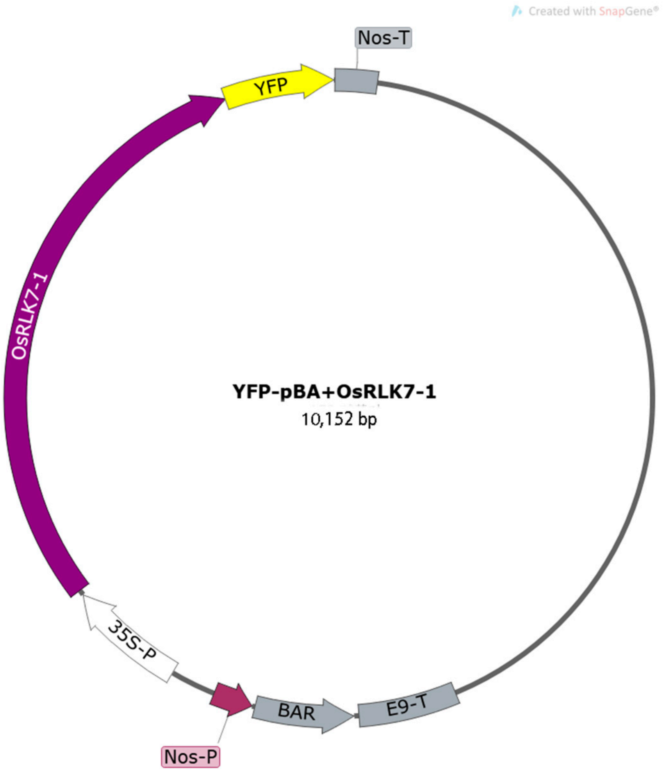


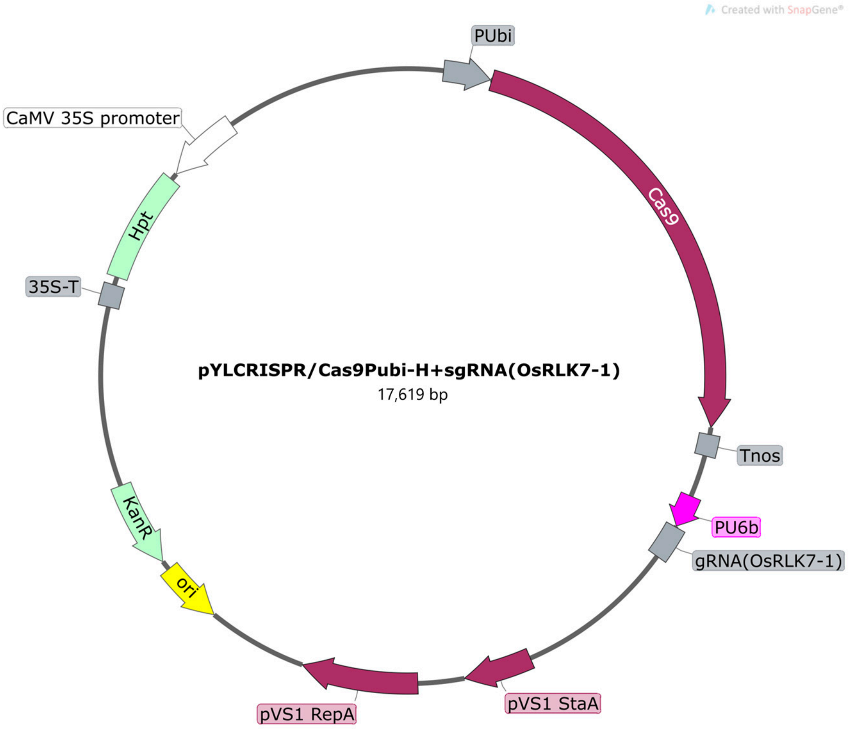


**Figure S9.** Vectors used in this study.

(a) The vector used for subcellular localization of OsRLK7-1. (b) The vector used for obtaining transgenic plants by pY-LCRISPR/Cas9Pubi-H.

**Table S1.** Primers used for qRT-PCR

| Name | TIGR ID | Forward primer (5′-…-3′) | Reverse primer (5′-…-3′) |
|---|---|---|---|
| *OsACTIN* | *LOC_Os03g50885* | TGGACAGGTTATCACCATTGGT | CCGCAGCTTCCATTCCTATG |
| *OsMPK3* | *LOC_Os03g17700* | CGACTTCGAGCAGAAGGCTCTA | GTTCATCTCGATCGCTTCGTT |
| *OsMPK6* | *LOC_Os06g06090* | CGCACGCTCAGGGAGATC | GGTATGATATCCCTTATGGCAACAA |
| *OsWRKY30* | *LOC_Os01g61080* | AGGCAAGCACAGCCATGAC | GAAGACGATACGTTGGCATTAGC |
| *OsWRKY33* | *LOC_Os01g53040* | AACGGCTGCTCCATGAAGAA | TTGTGTGCGCCCTTGTAGAC |
| *OsWRKY45* | *LOC_Os05g25770* | CGACCGGAAGGCCAACT | GAAGTAGGCCTTTGGGTGCTT |

**Table S2.** Primers used for PCR

| Name | TIGR ID | Forward primer (5′-…-3′) | Reverse primer (5′-…-3′) |
|---|---|---|---|
| *OsRLK7-1* | / | ATGCCACCGCCCTCCCTTCT | CTAGGGGATCACCTTGACCTTCACCGA |
| *KO-RLK7-1* | *LOC_Os12g43640* | ATGCCACCGCCCTCCCTTCT | CTAGGGGATCACCTTGACCTTCACCGA |
| Fusion RLK7-1&YFP | / | ACGCGTTTAATTAAGATGCCACCGCCCTCCCTTCT | TGACGTCCCGGGATCCGGGGATCACCTTGACCTTCACCGA |
| *Cas9*-test | / | GTTGAGCCGCACAGAACGTCGAA | AAACTTCGACGTTCTGTGCGGCT |
| *KO-RLK7-1-Cas9* region check | *LOC_Os12g43640* | AGGCTGCTGACCTACTTACTACC | CCGTCTTCTCGAAGAACCCAT |
| Off-target test 1 | *LOC_Os06g35770* | AGGAGGAGGAGGAGGAGGAGGAGGA | TCGGAGAAGGTGAACCCGAGGTCTT |
| Off-target test 2 | *LOC_Os01g55120* | GCCGCATTTCCGTCTCCATCC | CCCGCTCACCAAATCCATCTTCA |
| Off-target test 3 | *LOC_Os02g01740* | GTCTACAAGCCCCAGACCAA | AATTCAACAGCAACGCCGAT |
| Target site of *RLK7-1* for overlapping PCR | / | AGCCGCACAGAACGTCGAACAACACAAGCGGCAGC | AAACTTCGACGTTCTGTGCGGCTGTTTTAGAGCTAGAAAT |
| sgRNA expression cassette assembling | / | CTCCGTTTTACCTGTGGAATCG | CGGAGGAAAATTCCATCCAC |
| Pps / Pgs | / | TTCAGAGGTCTCTCTCGACTAGTATGGAATCGGCAGCAAAGG | AGCGTGGGTCTCGACCGACGCGTATCCATCCACTCCAAGCTC |
